# Fabrication and application of naturally sourced nano-pigments based on algal biomass in multifunctional coatings

**DOI:** 10.1038/s41598-025-97752-3

**Published:** 2025-05-02

**Authors:** Walaa M. Abd El-Gawad, Sayeda M. Abdo

**Affiliations:** 1 Polymers and Pigments Department, National Research Centre, Dokki, Giza, Egypt; 2 Water Pollution Research Department, National Research Centre, Dokki, Giza, Egypt

**Keywords:** Algal biomass, Naturally sourced nanopigments, Sustainable colored coatings, Antimicrobial activity and thermal stability, Biological techniques, Microbiology, Environmental sciences, Materials science

## Abstract

This work aims to synthesize economical and eco-friendly naturally sourced nano-pigments with bright colors, antimicrobial activity, and thermal stability from algal cells cultivated in wastewater, which are then harvested, dried, and converted into algal biomass (AB). Algal biomass (AB) was covered by a very thin nano-layer of either zinc ferrite or cerium ferrite, which does not exceed 10%. After the synthesis and characterization, these nanopigments were incorporated into alkyd resin in two proportions (2.5% and 5%). The antimicrobial activity and color of the produced coatings were investigated using the disc diffusion and CIELab methods, respectively. Besides, their thermal stability was examined using scanning electron microscopy (SEM) and thermal gravimetric analysis (TGA). The results of antimicrobial activity demonstrate that the effect of all coatings on fungi is greater than their effect on bacteria and that films containing 5% of nano-pigments gave an inhibition zone for microbes greater than those containing 2.5%. Additionally, the thermal stability results of the film containing algal biomass show very high weight loss, reaching 47.5% in group I and 76% in group II. While coatings containing zinc ferrite/AB and cerium ferrite/AB, weight loss doesn’t exceed 20%, and films containing 5% are the best.

## Introduction

Recently, pigments have significant industrial importance and find diverse applications in coatings, the paper industry, cosmetics, and the chemical industry^1^. Most of the pigments utilized in several industries are synthetic due to their high purity, stability, and consistency; besides, they can be customized for specific features depending on the application^2^.

One of the most important synthetic pigments that has received a lot of attention lately is ferrites, which are composite oxides containing ferric ions. MFe_2_O_4_ is their typical composition, where M is one of the divalent metal ions, such as Zn^2+^, Ce^2+^, Ni^2+^, Co^2+^, etc.^3–5^. Owing to their unique physicochemical features, spinel ferrites offer exceptional chemical and thermal stability, various sizes and forms, a large surface area, excellent electric and magnetic characteristics, and a wide range of applications in both science and technology^6–8^. Despite these advantages, there is an increasing trend in the industry to switch from these synthetic pigments to naturally sourced pigments, which creates a high demand for naturally sourced pigments. This may be due to the toxicity of synthetic pigments, their environmental and sustainability concerns, and the pollution potential of their production^9^. Thus, the employment of naturally sourced pigments has become a very crucial and trendy research topic^10^.

Recently, discovering novel bio-based sources of pigments and establishing their generation become an important way to increase the sustainability of the modern industry^11^. Microalgae are considered a sustainable source of natural pigments because they can be cultivated using sunlight, carbon dioxide, and nutrients without competing with traditional agriculture for land or freshwater resources^12,13^. They have a high photosynthetic efficiency and can be grown in closed systems, such as photo-bioreactors, which minimize water usage and prevent the release of pigments into the environment^14^. While microalgal pigments hold significant potential, there are still some challenges to address for their widespread adoption in the coating industry. These include optimizing pigment extraction methods, scaling up microalgae cultivation systems, ensuring consistent pigment quality, and cost competitiveness compared to synthetic pigments^15,16^.

On the other hand, naturally sourced pigments are usually more expensive, less stable, and more difficult to use compared to synthetic pigments, in addition to their limited range of hues. They are also more susceptible to color fading or deterioration because of their sensitivity to environmental factors such as heat, light, pH, oxygen, and different organic chemicals and metal ions that regulate their use^17,18^. The ongoing research and technological advancements are expected to overcome these hurdles and pave the way for the commercialization of microalgal pigments in coatings^16^.

This issue has prompted researchers to look for new ways to stabilize these naturally sourced pigments to facilitate their utilization. A wide range of approaches have been developed to address the aforementioned issues; the encapsulation approach demonstrates the greatest promise^17^. The main strategy of encapsulation is to entrap a core material within a wall material to assist in the delivery of an active agent to other media^19–21^. A possible route for the encapsulation of pigment is the use of the core–shell technique, in which a core of bio-based sources could be encapsulated by a shell of effective pigments^22^. Core–shell pigments have several advantages; they can help reduce expensive material consumption, besides their low toxicity^23,24^.

Additionally, the development of naturally sourced nano-pigments based on algal biomass through its chemical encapsulation with functionally stable and expensive pigments such as ferrites is trendy and an effective approach to overcome the disadvantages of the traditional extraction method. The key advantage of these naturally sourced pigments based on algal biomass is their ability to perform several functions, owing to their multi-composition^23–25^.

The novelty of this study is to prepare novel naturally sourced nanopigments to overcome the drawbacks of both natural and synthetic pigments by using core–shell technology. Herein, cheap and natural algal biomass (AB) is covered by nano layers of either cerium ferrite or zinc ferrite to be applied in multifunctional coatings. These synthesized pigments could offer bright colors, thermal stability, and antimicrobial activity in a single coat layer. Thus, the application of these sustainable pigments in one of the most strategic industries, which is the coatings industry, can serve both economic and environmental purposes.

## Experimental part

### Materials

Zinc sulfate, cerium sulfate, and ferric sulfate were obtained from LOBA in India with purities of 99, 99.99, and 98, respectively. Sodium hydroxide (NaOH) with 99.99% purity was obtained from Universal in India. Besides, cetyltrimethylammonium bromide (CTAB) was supplied from Adwic Co. in Egypt with 98% purity. Commercial alkyd resin, obtained from El Zain Chemicals Co. in Egypt, is a medium-oil-type solvent-based polyester. It is a soya-bean dehydrated castor oil resin consisting of 4% pentaerythritol, 30% O-phthalic anhydride, 12% pentaerythritol resinate, 4% glycerol, and 50% linseed oil.

### Pilot-scale high rate algal pond (HRAP)

A pilot-scale facility is being constructed to pave the way for upgrading larger-scale stabilization ponds. The selected site for this pilot is located at the Zenin Wastewater Treatment Plant in Giza, Egypt, with geographic coordinates 30°1′ 57.91″ N/31°10′53.53″ E. The site experiences an average daily temperature variation of 10°C and a seasonal temperature range of 15–40 °C.

The system under construction consists of the following components:

- An anaerobic pond
- A High Rate Algal Pond (HRAP)
- A solar dryer

The available land area for this pilot facility is 90 m^2^ (17 m × 14 m). The system is designed to treat 2,555 m^3^ of wastewater annually. The screened wastewater is fed into the anaerobic ponds, which have a hydraulic retention time of 5 days^26^.

### Community harvesting and enumeration in high-rate algal ponds

Different subsamples of the high-rate algal pond were collected at various depths. This sampling strategy allows for capturing the diversity of the algal community present in different regions of the pond. The collected subsamples were combined and mixed together. By blending the subsamples, a representative samples from the entire pond is obtained. After mixing, 0.2 ml of this sample was spread onto Sedgewick Rafter cells, which are specialized counting chambers. Identification of dominant algal species was conducted on five slides per sample, with each slide being examined and counted twice^27^. Algal identification was done using an Olympus X3 microscope (Olympus Corporation, Tokyo, Japan), following the keys to freshwater algae^28,29^.

Harvesting algal cells was carried out by collecting different subsamples of a high-rate algal pond at different depths. The sample was left to settle overnight. Allowing the sample to settle facilitates the separation of the algal cells from the water, as the cells tends to settle towards the bottom.

**Fig. 1:**
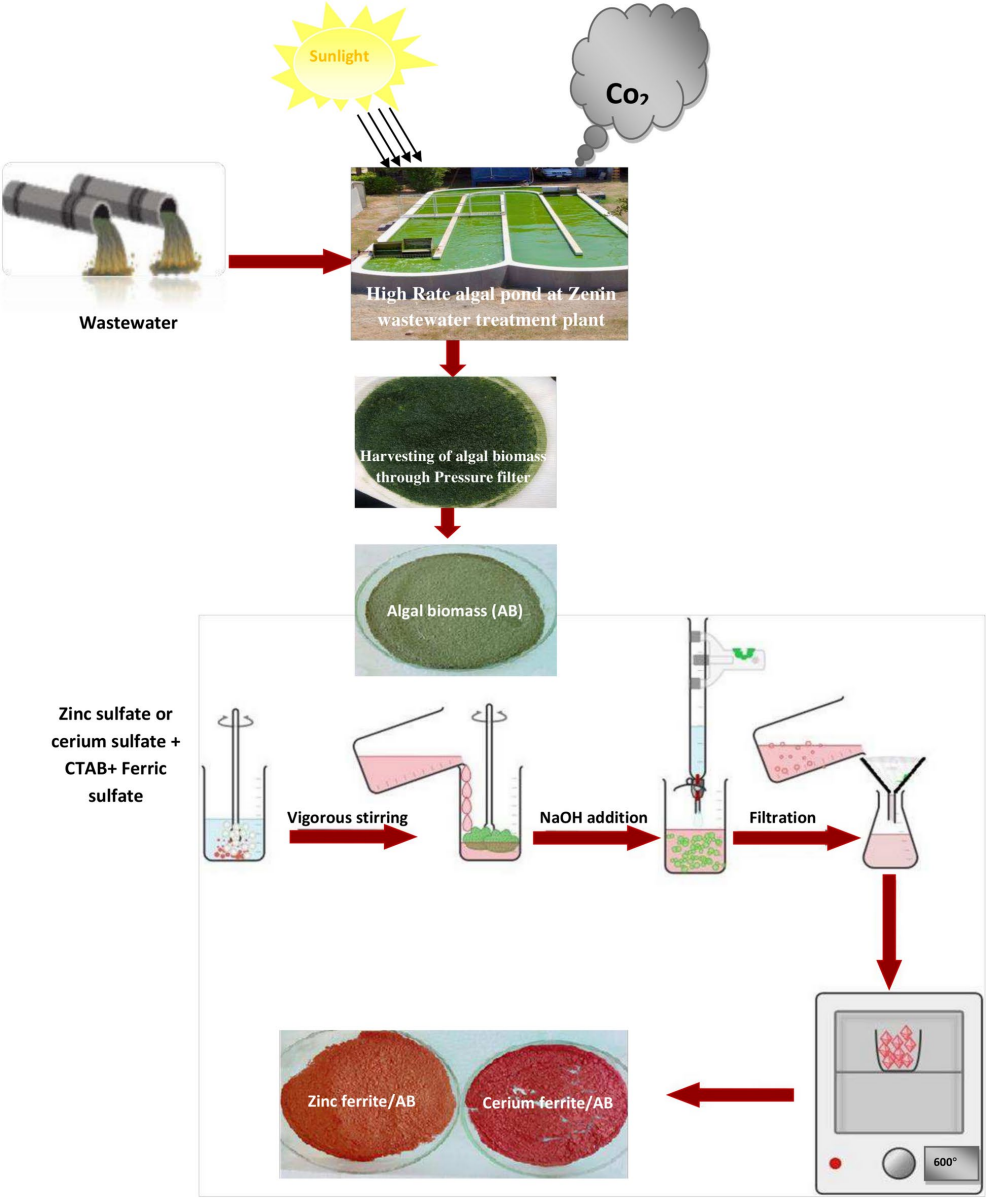
Synthesis process of proposed nanopigments.

Algal cells were collected through pressure filter presses to remove water from liquid wastewater residuals and produce a non liquid material referred to as algal “biomass” using a membrane polyester 550G/M2 10 micronas shown in Fig. 1. Algal biomass was then removed from the membrane and dried at 40 ºC.

The growth rate was assessed by determining the chlorophyll a content. The following equation was used for calculating the concentration of chlorophyll A (as µg/L)^26^.

$$\begin{gathered} {\text{C a}}\, = \,{11}.{85}\left( {{\text{OD664}}} \right) - {1}.{54}\left( {{\text{OD647}}} \right) - 0.0{8}({\text{OD63}}0) \hfill \\ {\text{Chlorophyll }}\;{\text{a}}\; \, \upmu {\text{g}}/{\text{L}}\, = \,{\text{Ca}} \times {\text{extract}}\;{\text{ volume }}/{\text{ volume}}\;{\text{ of }}\;{\text{sample}}\;{\text{L}} \hfill \\ \end{gathered}$$

Where: OD 664, 647 and 630 are the absorbance at 664,647 and 630.

For the purpose of grinding the algal biomass, a planetary ball mill (Retsch GmbH, Haan, Germany) that came equipped with a grinding jar and 1 mm beads made from yttrium-stabilized zirconium oxide was utilized. To ensure optimal performance and prevent overheating, the grinding process was paused every 10 min to allow the jar to cool. Following the grinding procedure, the beads were separated from the algal biomass using a sieve^30^.

### Cytotoxicity evaluation of algal biomass

An assessment was conducted between the MB and the human cancer cell line HePG 2 (Human hepatocellular carcinoma cell line). Sample concentrations between 20.000 and 312.5 μg/mL can be measured with the MTT test. Human tumor cell lines’ estimated cytotoxicity was verified using the MTT assay’s IC50 values and percentage of viable cells. 48 h at 37 °C and 5% CO_2_ were employed to test the biomass at dosages ranging from 1000 to 15.63 μg/mL. 50% of cells die within 48 h due to the sample’s lethal concentration (IC 50) of 1082 μg/mL. This proves there was no cellular harm from the algal biomass^31,32^.

### Synthesis of the naturally sourced nanopigments (zinc ferrite/AB and cerium ferrite/AB)

As shown in Fig. 1, 100 ml of 0.5 molar zinc sulfate or cerium sulfate was added with 100 ml of 1 molar iron sulfate and a 2.5% solution of CTAB, and this mixture was stirred well for an hour. After that, a solution of NaOH with a concentration of 5 mol/L was added drop by drop with continuous and rapid stirring until the pH of the mixture reached 9. After this step, the solution containing the precipitate was added to 100 g of green algal biomass drop by drop until the precipitate completely covered the surface of the biomass. Then the sediment was washed well with distilled water until the pH of the sediment reached neutral. To ensure that the sediment was completely cleaned and to make it easy to dry, it was washed with 99% ethyl alcohol. Then the sediment was dried at 80 °C to volatilize all the solvents and avoid petrification. After that, grinding the obtained sediment well was done to obtain a fluffy powder, and finally, these powders were calcined at 550 °C for two hours to form ferrite pigments^33,34^.

### Instrumental analysis methods

The structures and sizes of the produced naturally sourced nano-pigments were examined using transmission electron microscopy (TEM) and scanning electron microscopy/energy-dispersive X-ray analysis (SEM/EDX) methods using micro-analyzer electron probes (JEOL JX 1230 and JEOL JX 2840) in Japan, respectively. The ZPW388 particle sizing system obtained from Santa Barbara, California, USA, was used. A JASCO FTIR-4100 E FT-IR spectrometer (Japan) operating in absorption mode in the wave number range of 4000–400 cm^−1^ was used to measure the FT-IR spectra of the synthesized nano-pigments. UV–visible Spectrophotometer (Peak E-2100 uv, China) was used. The TGA-50 Thermal Analyzer (Schimadzu Co., Tokyo, Japan) was used to perform thermal gravimetric analysis (TGA).

### Paints preparation

Algal biomass, zinc ferrite/AB, and cerium ferrite/AB naturally sourced nanopigments were integrated into alkyd resin by a ball mill according to the proportion of nanopigments to alkyd resin mass fractions of 2.5% and 5%, respectively. The coatings were produced without the addition of other fillers to estimate the actual effect of the synthesized nanopigments. Firstly, a mixture of N-butanol and xylene with a weight ratio of 3:7 was made. After that, in group I, to prepare 100 g of paints, 2.5 g of algal biomass, zinc ferrite/AB, or cerium ferrite/AB were dispersed in almost 3 ml of the N-butanol and xylene mixture and then mixed together by ultrasonic for 0.5 h. In the case of group II, 5 g of algal biomass, or nanopigments, was dispersed in 5 ml of the N-butanol and xylene mixture and then mixed together by ultrasonic for 0.5 h to avoid agglomeration of nanopigments in the viscous alkyd. Then, the obtained solutions were mixed with the alkyd resin in the gridding machine (ball mill) for 1 h. After the mixing process, the obtained paints were filtered to remove any unmixed particles. Finally, six formulations based on alkyd containing algal biomass, zinc ferrite/AB, or cerium ferrite/AB, at 2.5% and 5%, were obtained. The surface of the steel with dimensions of 2*2 cm was prepared according to ASTM D609-00 (Procedure D: Solvent Wiping) to determine its thermal stability with a thickness of 120 µm. Plastic circles with a diameter of 2 mm were cut and painted to form thin films to test color and antimicrobial activity^35,36^. The formulations are mentioned in Table 1.

**Table 1:** The coatings formulation containing the naturally sourced nano-pigments.

Coating	Ingredients (Wt.%)			
	Group I			
	Epoxy	Algal biomass	Zinc ferrite/AB	Cerium ferrite/AB
Algal biomass	97.5	2.5	–	–
Zinc ferrite/AB	97.5	–	2.5	–
Cerium ferrite/AB	97.5	–	–	2.5

Coating	Ingredients (Wt.%)			
	Group II			
	Epoxy	Algal biomass	Zinc ferrite/AB	Cerium ferrite/AB
Algal biomass	95	5	–	–
Zinc ferrite/AB	95	–	5	–
Cerium ferrite/AB	95	–	–	5

### Physico-mechanical properties

Different physico-mechanical properties were assessed using a number of ASTM criteria, including impact resistance (ASTM D 2794), which is responsible for the ability of coats to resist impact force, and ductility (ASTM D 5638), which is used to determine the elasticity and flexibility of the coated films. Moreover, hardness (ASTM D 6577) is used to investigate the strength and stiffness of the films. Additionally, pull-off strength (ASTM D 4541) is used to determine the adhesion of coated films.

### Color and gloss measurements

The color of the synthesized nano-pigments was investigated using the Lovibond Tintometer RT 100 Color by the CIELab method. In this test, L* is the lightness axis (0 for black and 100 for white). a* is the green (−) to red (+) axis, and b* is the blue (−) to yellow (+) axis. The gloss test, which was carried out as mentioned in ASTM D1455, is a significant tool to investigate surface appearance. It’s a standard test procedure for investigating the specular gloss. Using a "gloss meter," a direct measurement was made. Measurements are usually done on a clean, straight, and uniform surface. Although a tiny area, such as 1 cm^2^, can be used for the measurement, it is best to utilize a larger surface to guarantee that the sample’s surface is homogeneous and that the results are representative^33^.

### Antimicrobial measurements

Qualitative antimicrobial estimations of the produced coatings were performed on nutrient agar plates according to literature^37^. The inoculation of *Staphylococcus aureus* and *Candida albicans* was prepared from fresh overnight broth cultures using nutrient broth medium that was incubated at 37 °C^38^. These pathogenic strains were generated with their inoculum size adjusted to about 1.5 × 108 CFU/ml (0.5 McFarland standards)^39^. In all plates containing 20.0 ml of the sterile nutrient agar medium, 2.5 µl of the yeastal and bacterial inocula were added^40^. The coatings were applied to the inoculated agar plates that had been previously produced using the disc diffusion method after the media had cooled and solidified. The inoculated plates were put in a refrigerator for 1 h for diffusion^41^. After that, the incubation at 37 °C for 24 h occurred, and the zones of inhibition were determined in mm. The trials were repeated three times for each antimicrobial test, and the standard deviation was computed^25^.

### Thermal stability evaluation techniques

The thermal gravimetric analysis of coats was investigated by the Perkin Elmer thermogravimetric analyzer TGA7 technique (USA). Additionally, SEM with the model JEOL JX 2840, Japan, was utilized to explore the scanned surfaces before and after exposure to heat according to ASTM D2485. To reach the final temperature, the heat was steadily raised by 55 °C with alternate time intervals, and the following describes the temperature cycle over time: [205 °C for 8 h, 260 °C for 16 h, 315 °C for 8 h, 370 °C for 16 h, 425 °C for 8 h, 480 °C for 16 h, and 525 °C for 8 h]^30^.

## Results and discussion

### Identification of algal community structure

**Fig. 2:**
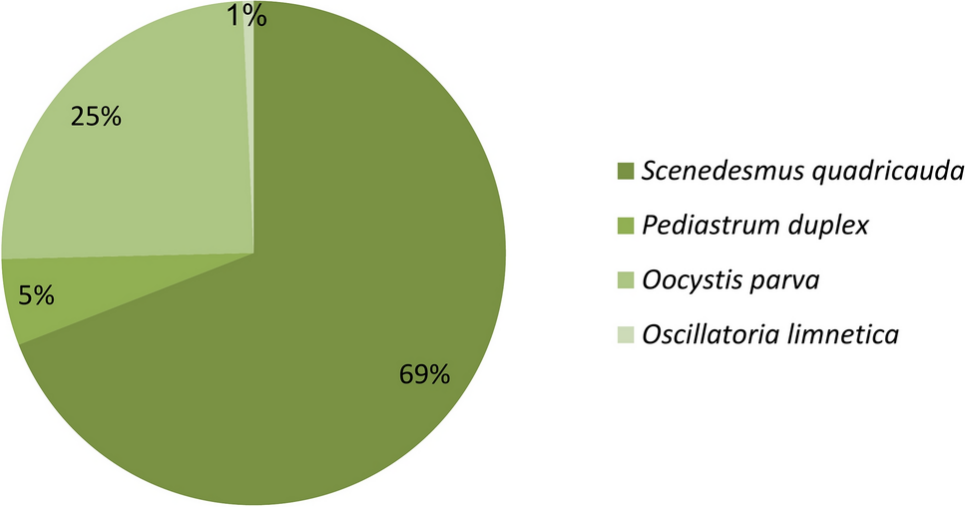
Algal community structure in High Rate Algal Pond (HRAP).

**Fig. 3:**
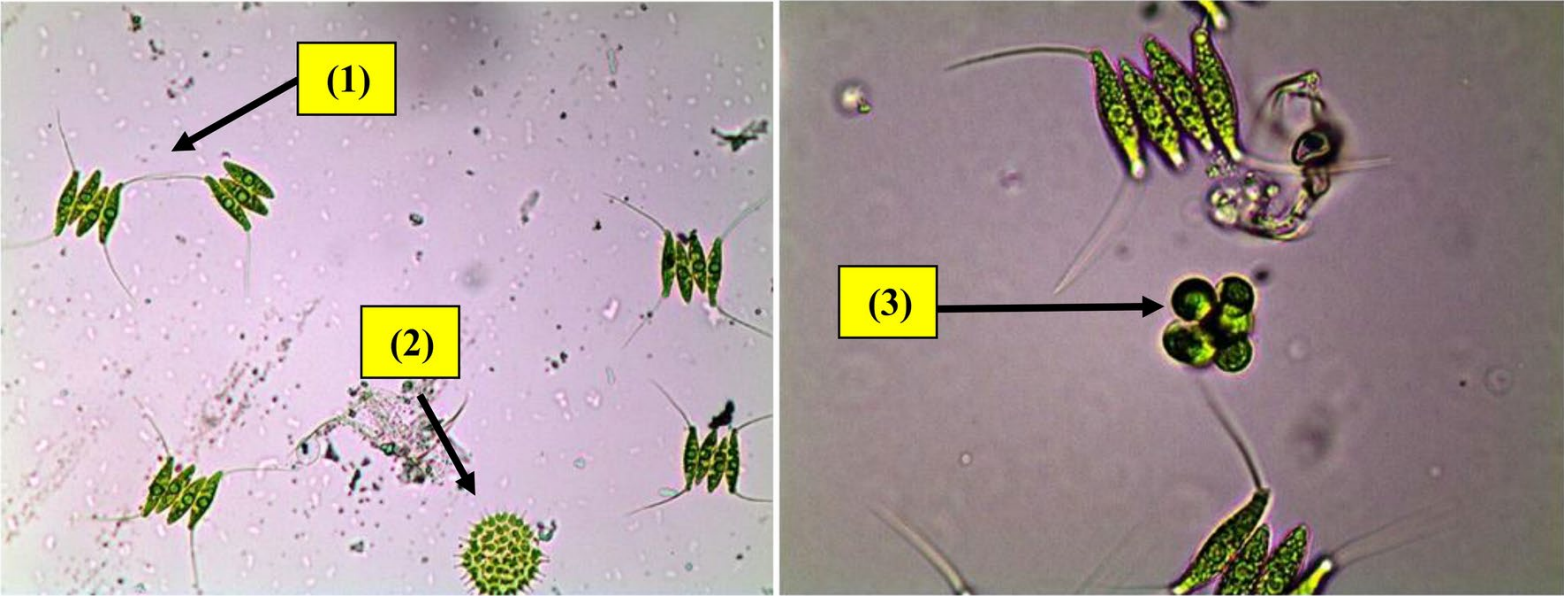
Microalgal species detected in samples collected from a high-rate algal pond (1)* Scenedesmus quadricauda*, (2)* Pediastrum duplex* and (3)* Oocystis parva.*

Samples collected were dominated by *Scenedesmus quadricauda*, a genus of green microalgae that is often found in freshwater environments. It was found in water samples collected from high-rate algal ponds in a percentage of 69%, as revealed in Fig. 2. *Oocystis parva* is characterized by its spherical or oval-shaped cells and is known to form colonies. It is another green microalga commonly found in collected samples with a percentage of 25%, as shown in Fig. 3. *Pediastrum gracillimum*, also belonging to the Chlorophyta group, is a colonial green microalga characterized by its star-shaped appearance. In addition, *Microcystis aeruginosa* and *Oscillatoria limnetica*, which belong to the blue-green microalgae group, were rarely detected. The presence of these microalgae in the high-rate algal pond indicates their adaptability to the wastewater treatment conditions provided in the pond. These microalgae play a crucial role in the pond’s ecosystem by photosynthesizing and converting nutrients, such as nitrogen and phosphorus, into biomass. This helps in the removal of pollutants.

The chlorophyll content is commonly used as an indication of the growth condition and biomass of the algal community structure in high-rate algal ponds. The chlorophyll (Chl) family are essential pigments required by all photosynthetic organisms to absorb light energy and play a key role in acclimation to environments with a varied light spectra^42^. Chlorophyll is essential for photosynthesis, which is the process by which algae convert sunlight into energy. Chlorophyll A content monitoring in a high-rate algal pond was performed, and the result indicates that chlorophyll A content reached 2.6 mg/L at the time of harvesting algal biomass.

By measuring the chlorophyll content, researchers can estimate the abundance and productivity of algae in a high-rate algal pond. Higher chlorophyll concentrations generally indicate a higher density of algae and more active photosynthesis, suggesting favorable growth conditions. Conversely, lower chlorophyll concentrations may indicate suboptimal conditions or nutrient limitations that can affect algal growth^43^.

Monitoring chlorophyll levels over time can provide insights into the dynamics of algal blooms, nutrient availability, and the overall health of the algal community in high-rate algal ponds^44^. This information is crucial for optimizing pond management strategies, such as nutrient dosing, light exposure, and harvesting schedules, to achieve desired treatment outcomes and maximize biomass production for various applications, including wastewater treatment and biofuel production.

### Characterization of the synthesized naturally sourced nanopigments

#### Scanning electron microscopy (SEM) and transmission electron microscopy (TEM)

**Fig. 4:**
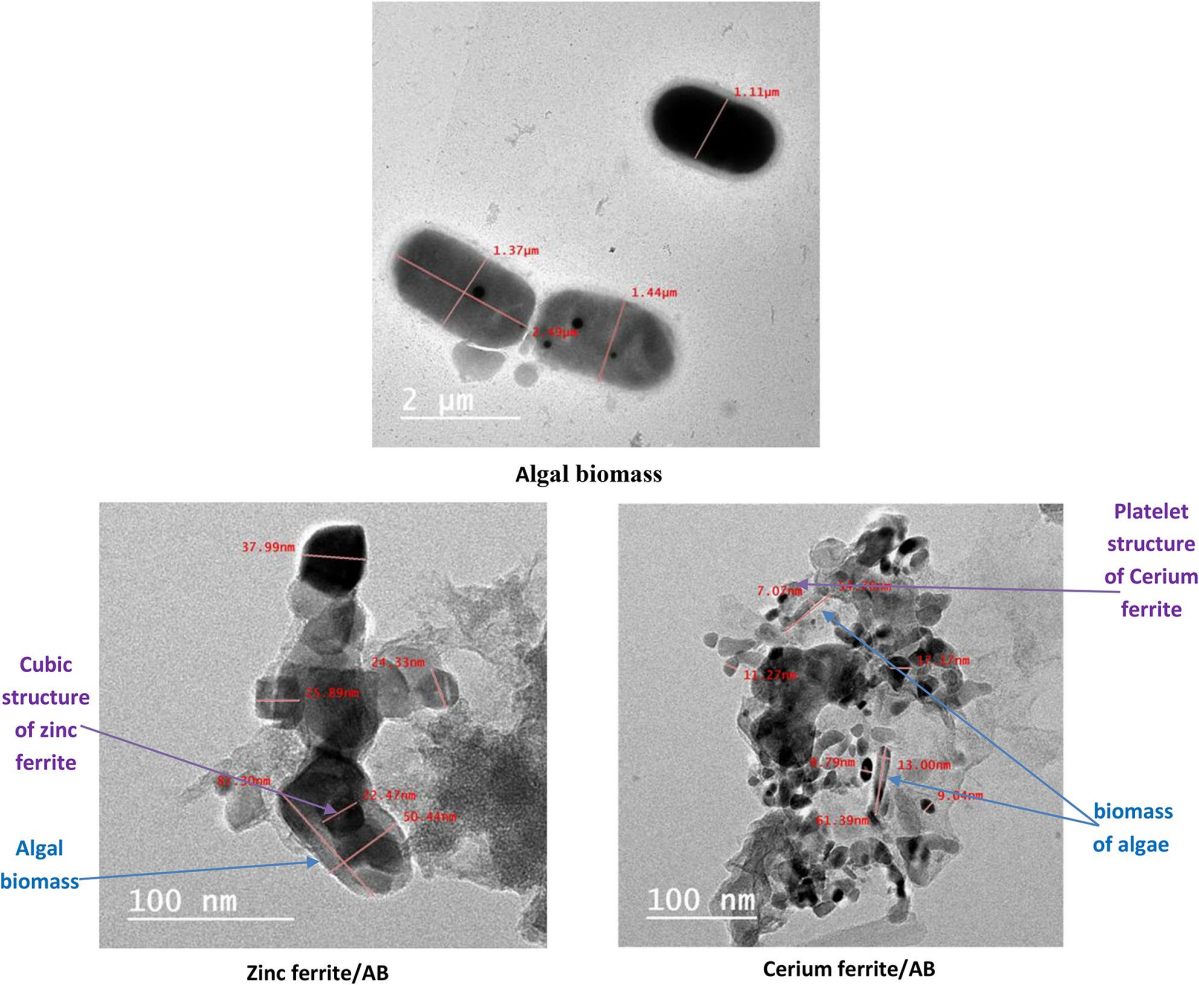
TEM of algal biomass, zinc ferrite/AB and cerium ferrite/AB.

**Fig. 5:**
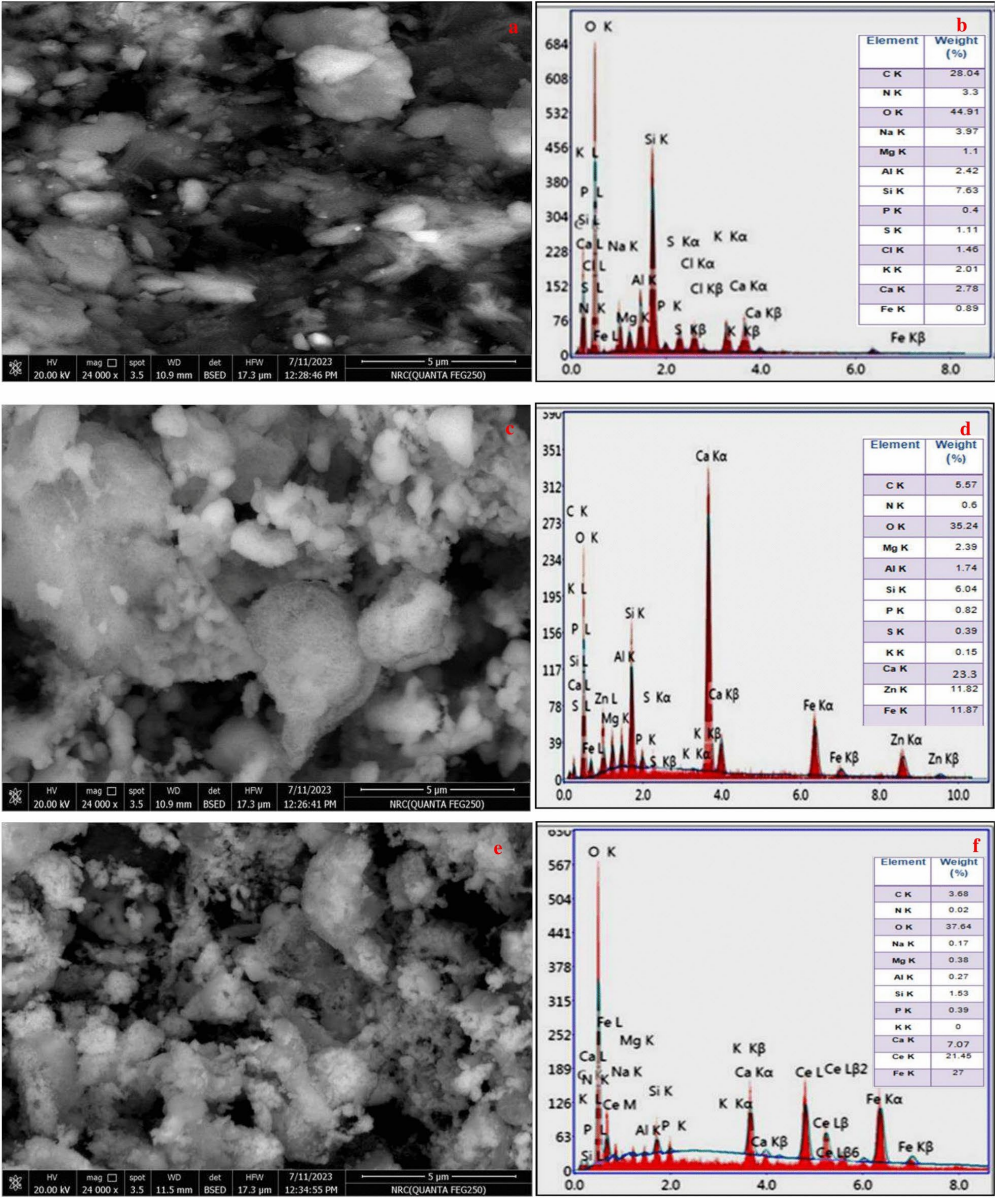
SEM/EDX of (**a**, **b**) algal biomass, (**c**, **d**) zinc ferrite/AB and (**e**, **f**) cerium ferrite/AB, respectively.

In Figs. 4, 5, TEM and SEM photos express the shape and size of algal biomass particles without treatment and the synthesized naturally sourced nanopigments. The images showed the presence of algal biomass grains in the form of a micron-sized cell. In the case of the prepared nanopigments, algal biomass particles appeared surrounded and covered with either zinc ferrite or cerium ferrite particles. Whereas, in the case of zinc ferrite/AB, the algal biomass particles appeared surrounded and covered with zinc ferrite particles, which appeared in the form of nano-sized cubes, while in the case of cerium ferrite/AB nanopigment, nano-sized, plate-shaped cerium ferrite particles appeared surrounding algal biomass particles. These images well express the encapsulation of algal biomass with a thin layer of ferrites, which is proof of the successful preparation of naturally sourced nanopigments.

#### Energy dispersive X-ray analysis (EDX)

Figure 5 shows the EDX analysis of algal biomass and the synthesized nanopigments. This analysis determines the elements present on the surface of the sample up to a depth of one micron. Therefore, this analysis is usually used to prove the deposition of a very thin layer of expensive pigments on the surface of other cheap materials. The figure indicates that the algal biomass used contains several elements such as oxygen, calcium, phosphorus, silicon, sulfur, nitrogen, carbon, potassium, sodium, aluminium, magnesium, chlorine, and a small percentage of iron. Additionally, Fig. 5 exhibits the presence of zinc and an increase in the percentage of iron due to the deposition of the zinc ferrite layer on the surface of the algal biomass, in addition to the appearance of other elements such as oxygen, calcium, phosphorus, silicon, potassium, aluminum, and magnesium, which are already present in the algal biomass used. Also, in the cerium ferrite/AB nano-pigment, both cerium and iron appeared in a high percentage with the rest of the elements present in the algal biomass, as shown in Fig. 5.

#### Zeta potential

**Fig. 6:**
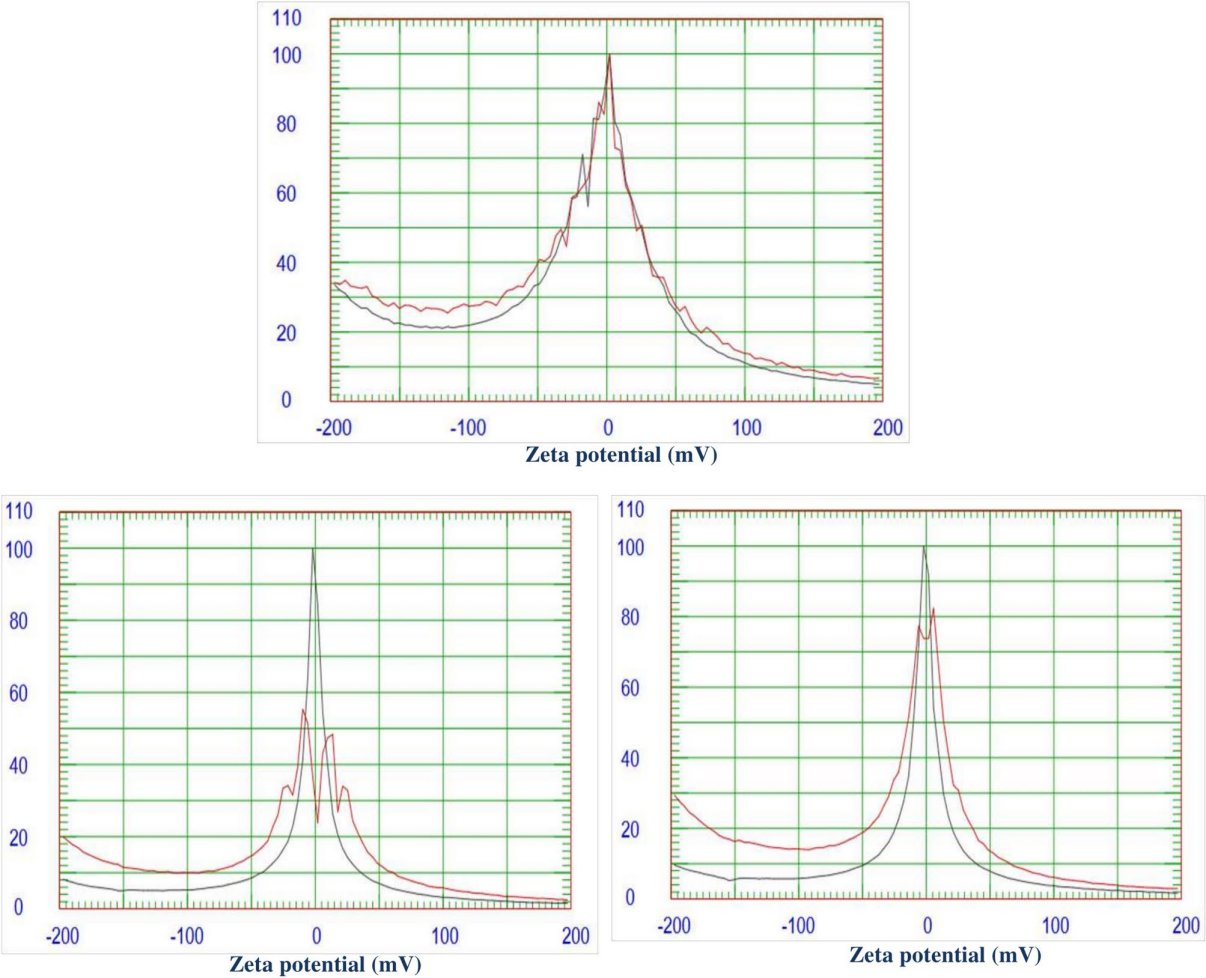
Zeta potential of algal biomass, zinc ferrite/AB and cerium ferrite/AB.

The zeta potential of algal biomass and the synthesized naturally sourced nanopigments is shown in Fig. 6. It is commonly known that zeta potential can shed light on whether nanoparticles will remain stable in the medium in which they are distributed. Through electrostatic repulsion, nanoparticels can be stabilized at zeta potential values >  ± 30 mV. It is noteworthy that nanoparticles with a zeta potential of less than 30 mV can also be sterically stabilized^45–47^.

The figure illustrates that, under the intended usage conditions, algal biomass does not agglomerate and falls within the defined range, indicating its stability as its average zeta potential is − 47.78 mV. In the case of both zinc ferrite/AB and cerium ferrite/AB nanopigments, the results obtained indicate that they are less than ± 30 mV, as their zeta potential values are − 19.7887 mV and − 29.87 mV, respectively. The value of cerium ferrite/AB is better than that of zinc ferrite/AB, which indicates that zinc ferrite/AB has the ability to agglomerate easily. For this reason, during the preparation of paints, the nanopigments were dispersed in the solvents firstly using ultrasonic before the mixing step in the ball mill.

### Fourier-transform infrared spectroscopy (FT-IR)

**Fig. 7:**
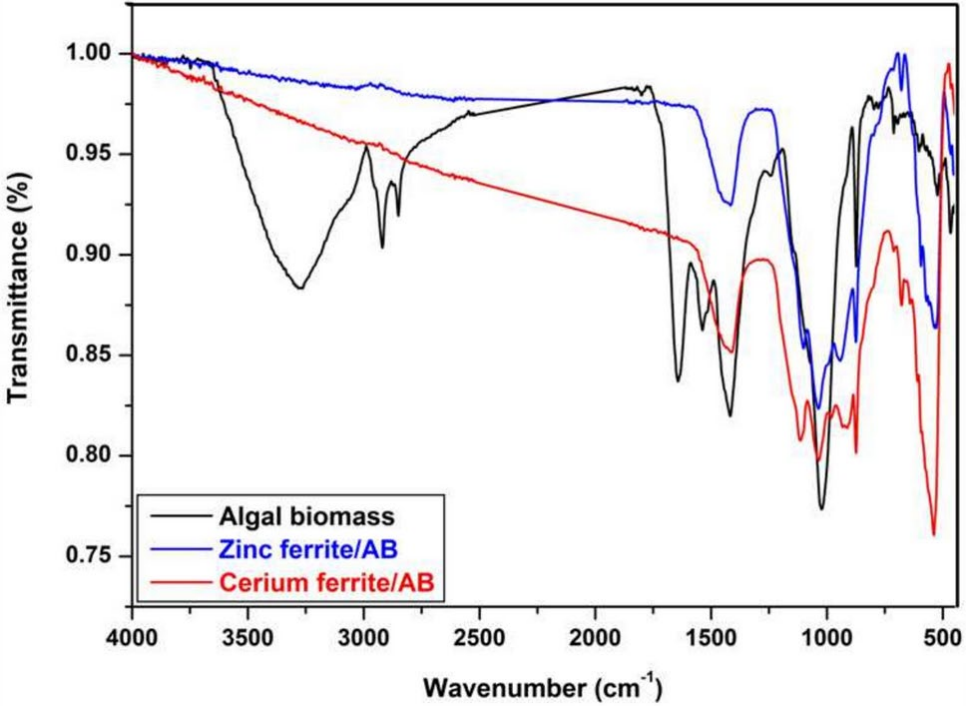
IR of algal biomass, zinc ferrite/AB and cerium ferrite/AB.

Figure 7 and Table 2 show the FTIR of algal biomass and the synthesized naturally sourced nanopigments. In the algal biomass, peaks are observed at various wavenumbers, such as 3200 cm^−1^ which is attributed to normal OH stretching, and 2926–2928 cm^−1^ which is related to methylene C–H stretching. Also, the peaks that appeared at 1660 and 1605 cm^−1^ are attributed to alkenyl C=C stretching and conjugated C=C, respectively. The appearance of peaks at 1440 cm^−1^ is due to methyl C–H asymmetric bending, respectively. Moreover, C–OH stretching is related to the peak at 1071^48^. A strong P–O–C stretching band can be detected by a 920 cm^−1^ band, while weak absorption bands between 580 and 670 cm^−1^ are caused by C–S and C=S stretching bands (sulfides)^45,49^.

**Table 2:** IR bands of algal biomass and the naturally sourced nano-pigments.

Peak (cm^−1)^	Algal biomass	Zinc ferrite/AB	Cerium ferrite/AB
3200	OH stretching	–	–
2928	Methylene C–H stretching	–	–
1660	Alkenyl C=C stretching	–	–
1605	Conjugated C=C	–	–
1440	Methyl C–H asymmetric	Methyl C–H asymmetric	Methyl C–H asymmetric
1071	C–OH stretching	C–OH stretching	C–OH stretching
920	P–O–C stretching	P–O–C stretching	P–O–C stretching
670	C=S stretching	C=S stretching	C=S stretching
580	C–S stretching	–	–
582	Tetrahedral Fe–O Stretching (Very weak)	Tetrahedral Fe–O stretching (strong)	Tetrahedral Fe–O stretching (strong)

In the case of naturally sourced nano-pigments, a strong characteristic band of tetrahedral Fe–O stretching for ferrites appeared at 582^51^. Moreover, the disappearance of bands from 2900–3400 cm^−1^ in the case of nano-pigments may be due to the encapsulation of algal biomass with a thin layer of ferrites.

### UV–visible spectroscopy

**Fig. 8:**
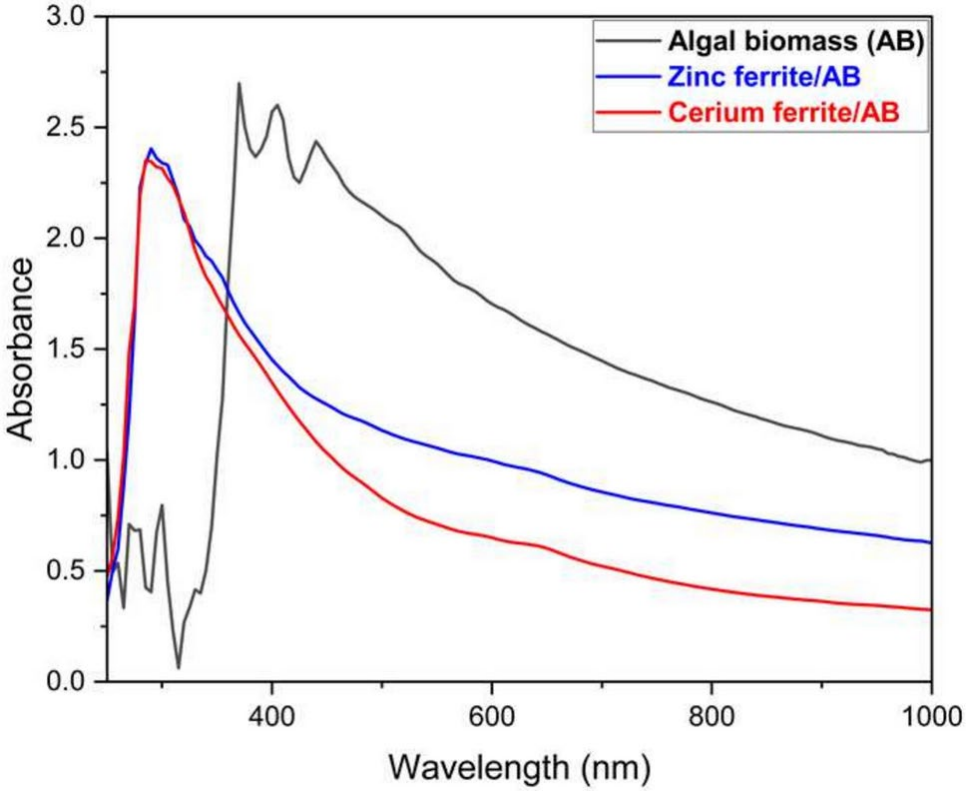
UV–Vis spectrum of algal biomass, zinc ferrite/AB and cerium ferrite/AB.

The UV–visible graph of algal biomass and the synthesized naturally sourced nanopigments is depicted in Fig. 8. It is well-known that the absorption spectrums of very small nanoparticles, which are usually less than 20 nm, are in the 300–350 nm range. Besides, when the particle size increases, redshift is noticed because of the light scattering process^52^. The figure shows that both zinc ferrite/AB and cerium ferrite show one sharp absorption band at almost 300 nm, which proves the small particle size of them. However, algal biomass shows a sharp band at 300 nm, besides three bands in the range of 330–440 nm due to the presence of larger particles.

### Thermal gravimetric analysis (TGA)

**Fig. 9:**
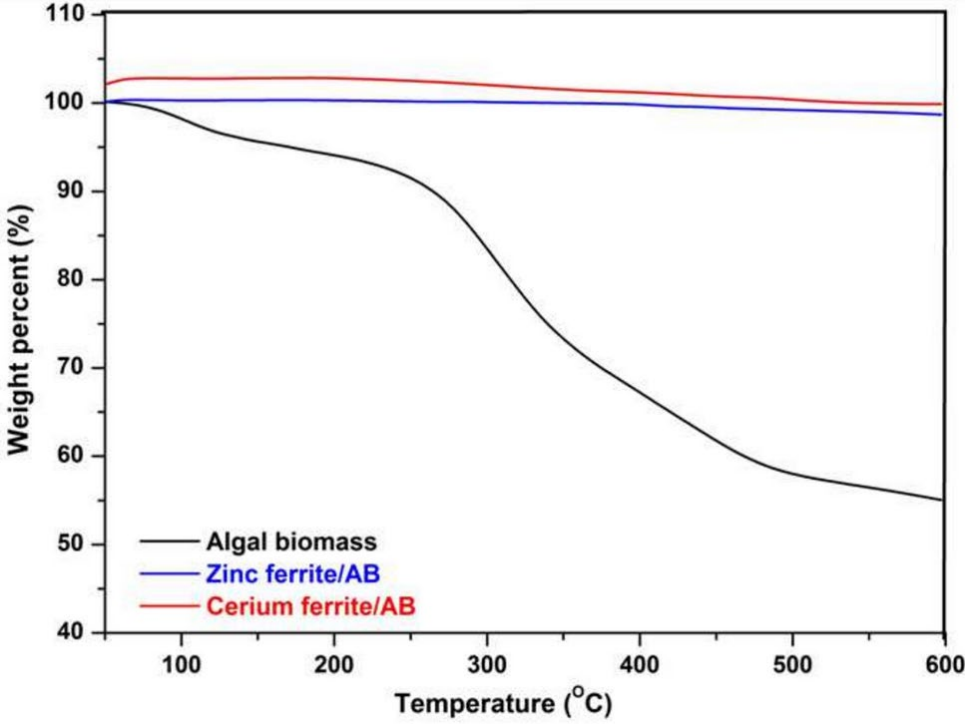
TGA of algal biomass, zinc ferrite/AB and cerium ferrite/AB.

Thermogravimetric behavior of algal biomass and the synthesized naturally sourced nanopigments is elucidated in Fig. 9. The TGA curve of algal biomass showed that there are three stages for its thermal degradation. The first degradation occurred from 30 to 200 ºC with a slight weight loss. This degradation may be due to the evaporation of water in the internal cells and the production of carbon oxides. In the second stage (200–500 ºC), the weight loss is high, which is attributed to the pyrolysis process occurring during the decomposition of algal biomass. Besides, most of the volatile organic compounds are released at this stage. In the third stage (500 to more than 600 ºC), a slightly low loss in weight is noticed. This loss clarifies that the carbonaceous matter in the residue is continuously decomposed until it reaches an asymptotic value^53^.

Additionally, the figure shows that the synthesized naturally sourced nanopigments (zinc ferrite/AB and cerium ferrite/AB) are thermally stable, which confirms that the encapsulation of the algal biomass with both nanopigments has generated thermally stable pigments. Zinc ferrite and cerium ferrite are types of spinel ferrites that belong to complex inorganic pigments, as their kind is two mixed oxides (for example, zinc ferrite is a mix of ZnO and Fe_2_O_3_, which order them to form the spinel ferrite type). These types of pigments are calcined at high temperatures to form their spinel structures. Therefore, ferrite pigments exhibit high thermal stability^54,55^.

### Coatings evaluation

#### The mechanical characteristics

**Fig. 10:**
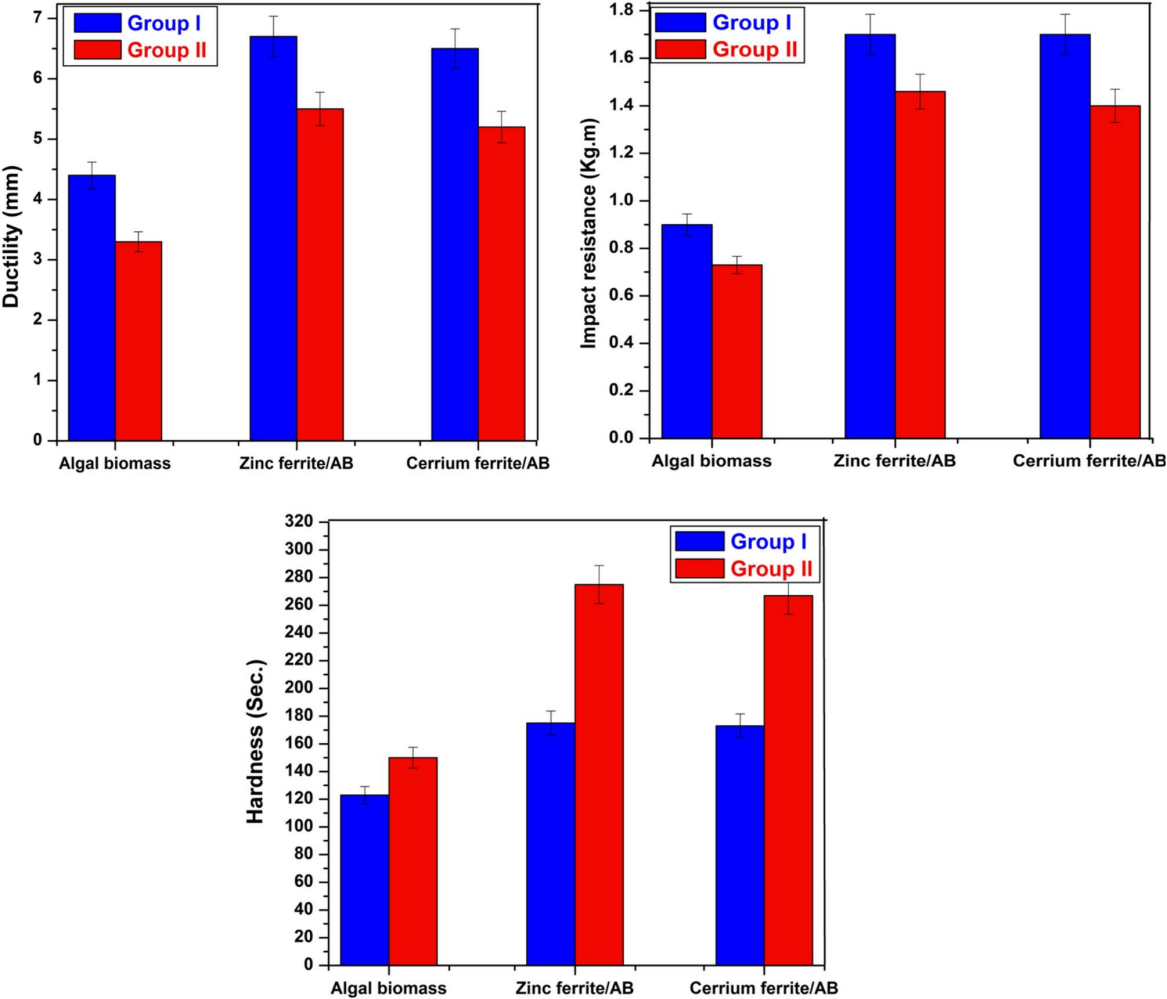
Mechanical properties of the prepared coatings.

Figure 10 shows the mechanical characteristics of coatings containing algal biomass and naturally sourced nanopigments. The findings show that the hardness of coatings containing naturally sourced nanopigments is higher than that of those containing algal biomass. The hardness of coatings containing AB, zinc ferrite/AB, and cerium ferrite is 150, 275, and 280 s. in group II and 122, 180, and 180 s. in group I, respectively. This may be due to the presence of zinc ferrite or cerium ferrite particles overlapped and surrounded by algal biomass particles, and these different particles have different sizes and shapes, which could help in the good arrangement of them in the polymeric matrix. In the case of algal biomass, its particles are large and on the micron scale, which couldn’t arrange them well and thus could leave voids in the polymeric matrix that led to the speedy cracking of the films. Moreover, the hardness of coatings in group II, which contains 5% algal biomass and nanopigments, is better than that in group I. This could be explained by the well-distributed nanopigments in the alkyd matrix, which form dense, compact films devoid of holes and gaps, making them tough and difficult to break down^25^. In the case of group I, the low content of solid materials could form voids that could lead to the films easily cracking.

Also, the figure shows that the impact resistance and ductility of group I are slightly higher than those of group II, and this may be related to the fact that the good distribution of the solid particles in between the alkyd chains can reduce their flexibility due to the restriction of particle movement^56^.

#### Pull-off strength results

The pull-off test was applied in this instance to investigate the adhesive strength of films containing algal biomass and nanopigments in dry and wet states. The values for adhesion loss (ψ) were estimated using the equation:

$${\uppsi } = \left( {{\upalpha }_{{\text{D}}} - {\upalpha }_{{\text{W}}} } \right)/{\upalpha }_{{\text{D}}} {*}100$$

where α_w_ represents the wet adhesion after immersion in water and αD indicates the dry adhesion.

**Fig. 11:**
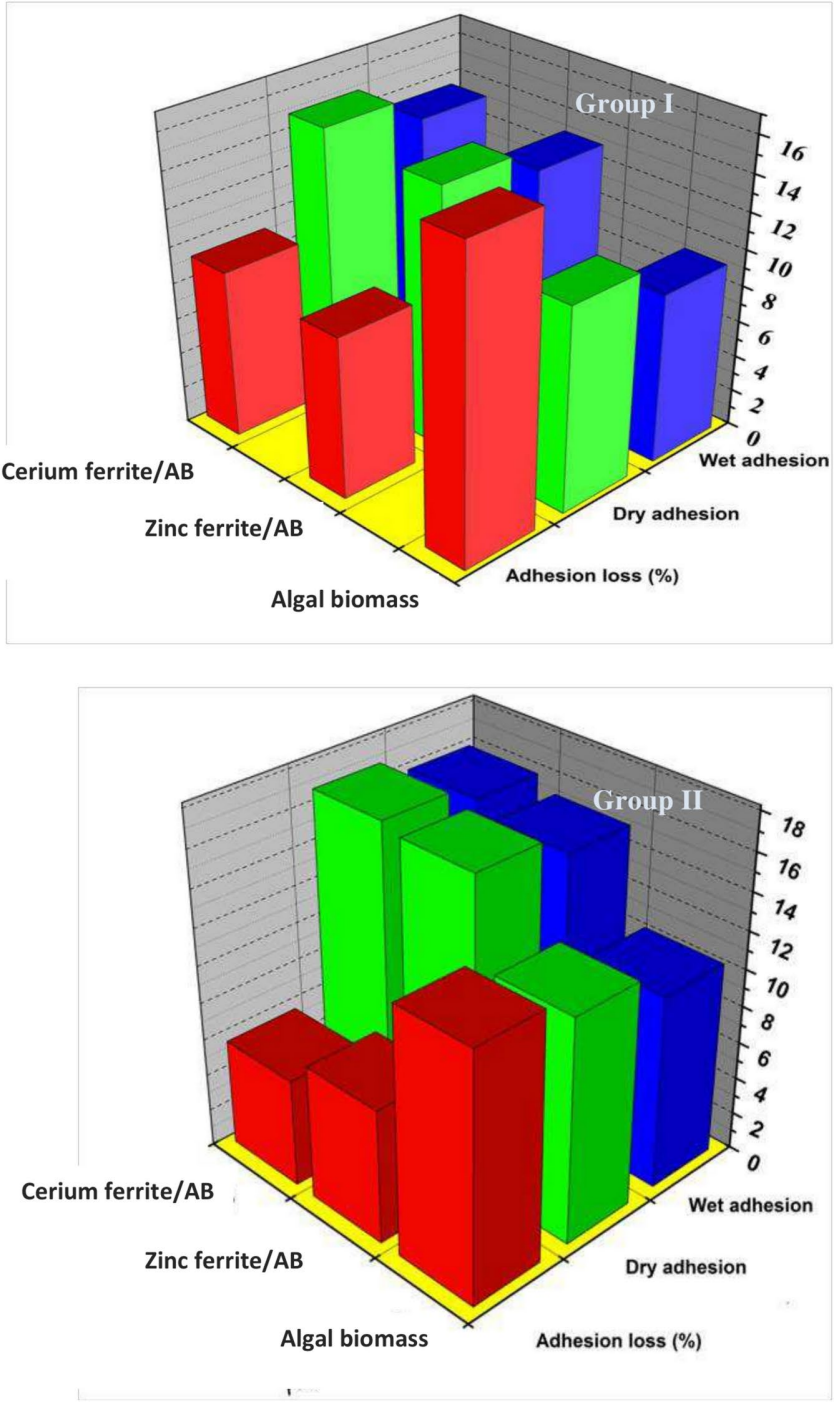
Pull-off test of the prepared coatings.

Figure 11 shows that the films based on algal biomass had the lowest adhesion strengths with the highest adhesion loss with value 13%, which is due to the micro-sized algal biomass particles that could lead to random order of the particles, causing the generation of voids that can negatively affect the adhesion strength.

In the case of coatings containing zinc ferrite/AB and cerium ferrite/AB, strong dry and wet adherence and minimal adhesion loss were observed. The adhesion loss ratios of coatings containing zinc ferrite/AB and cerium ferrite/AB are 8%. This may be due to the good packing of the different particles of both nano-ferrites and algal biomass; thus, the cohesion strength of the employed coatings is excellent^33,57^. Moreover, the adhesion of films containing high concentrations of algal biomass and nanopigments is greater than that of those based on low concentrations. As the existence of nanopigments in the alkyd with a high percentage could close all gaps between the chains that may facilitate the diffusion of water, adhesion is enhanced^25,58^.

#### The color and gloss measurements

**Fig. 12:**
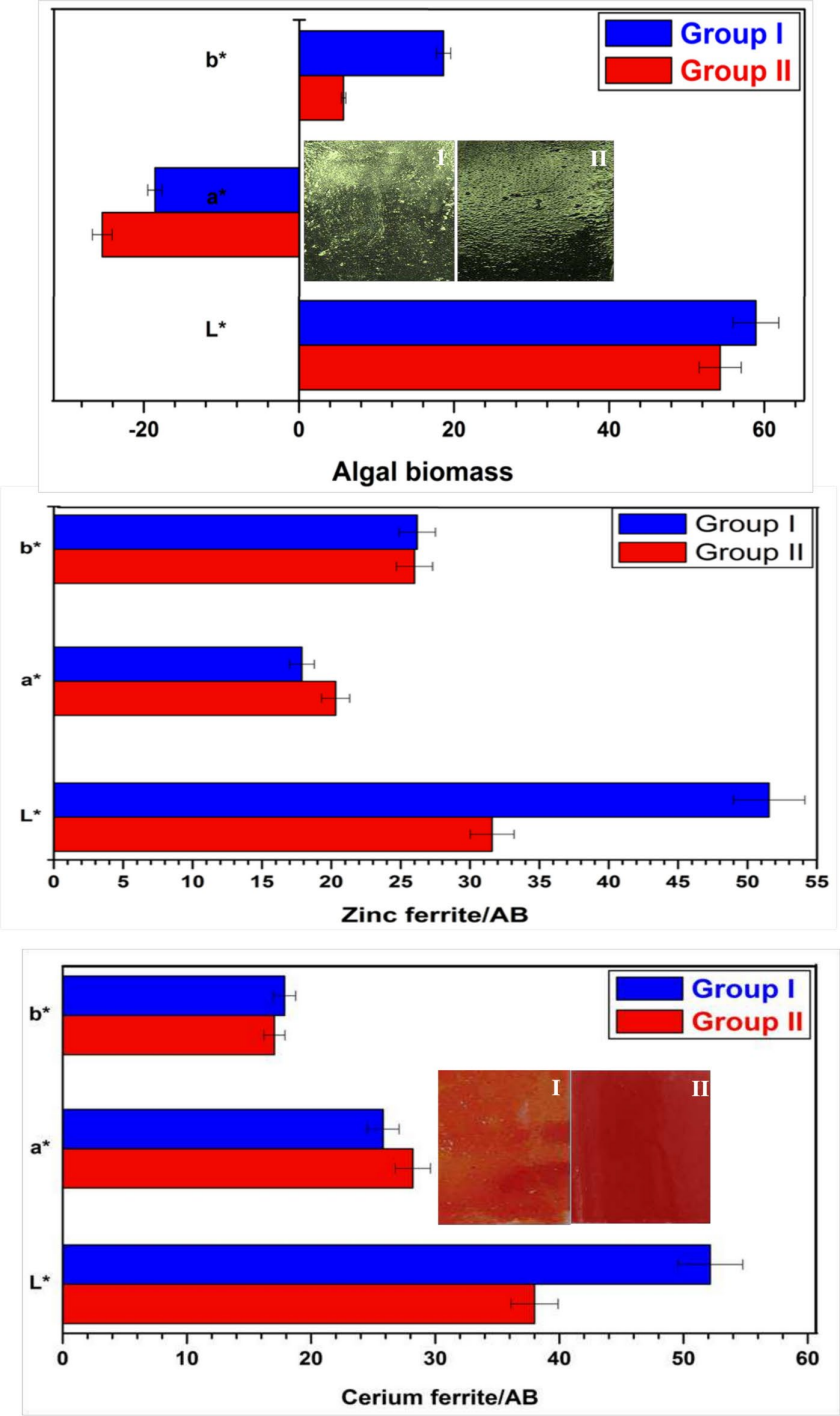
Color measurements of coatings containing algal biomass, zinc ferrite/AB and cerium ferrite/AB in both groups.

Figure 12 exhibits the color findings of coatings containing both algal biomass and naturally sourced nanopigments**.** In this test, L* is the lightness axis (0 for black and 100 for white), a* is the green (−) to red (+) axis, and b* is the blue (−) to yellow (+) axis. The results show that the films based on green algal biomass have (−a) values due to their green color. Besides, the film containing 5% (group II) has a (−a) value greater than that of the film containing 2.5% (group I), and this is evidence that the green color of the film containing 5% algal biomass is stronger than that of the film containing 2.5%. In the case of films containing both zinc ferrite/AB and cerium ferrite/AB, it was found that the (+ a) values are observed due to the red color. Additionally, the (+ a) values in 5% are higher than 2.5%, and these observations prove that the red color in group II is darker than in group I; thus, the intensity of the color increases with the increase in the percentage of nanopigment in the films. Furthermore, it was found that the value of (L*) decreases for the whole film with increasing the concentration of algal biomass and nanopigments in coatings because the coatings visible light absorption increases with increasing the nanopigment concentration, and the coatings light components (L*) reduce, thus increasing the red and green color hue^59^.

**Fig. 13:**
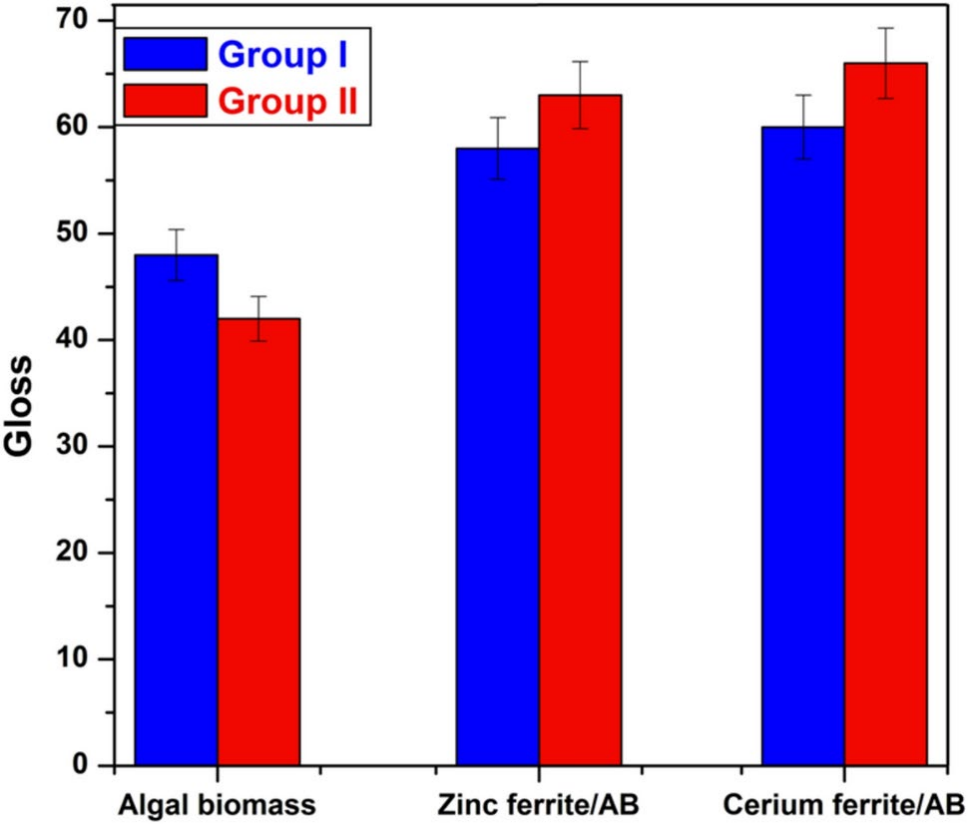
Gloss of the prepared coatings.

Furthermore, the results in Fig. 13 demonstrate that the gloss of the film has 2.5% algal biomass, which is higher than that of the film with 5% algal biomass due to the high percentage of alkyd in group I. In contrast, in the case of coatings containing zinc ferrite/AB and cerium ferrite/AB, the gloss of films with 2.5% is lower than films with 5%. This is due to the high concentration of nano-pigments in the polymeric matrix, which could lead to an enhancement of the metallic luster, thus increasing their gloss.

#### Antimicrobial evaluation

**Fig. 14:**
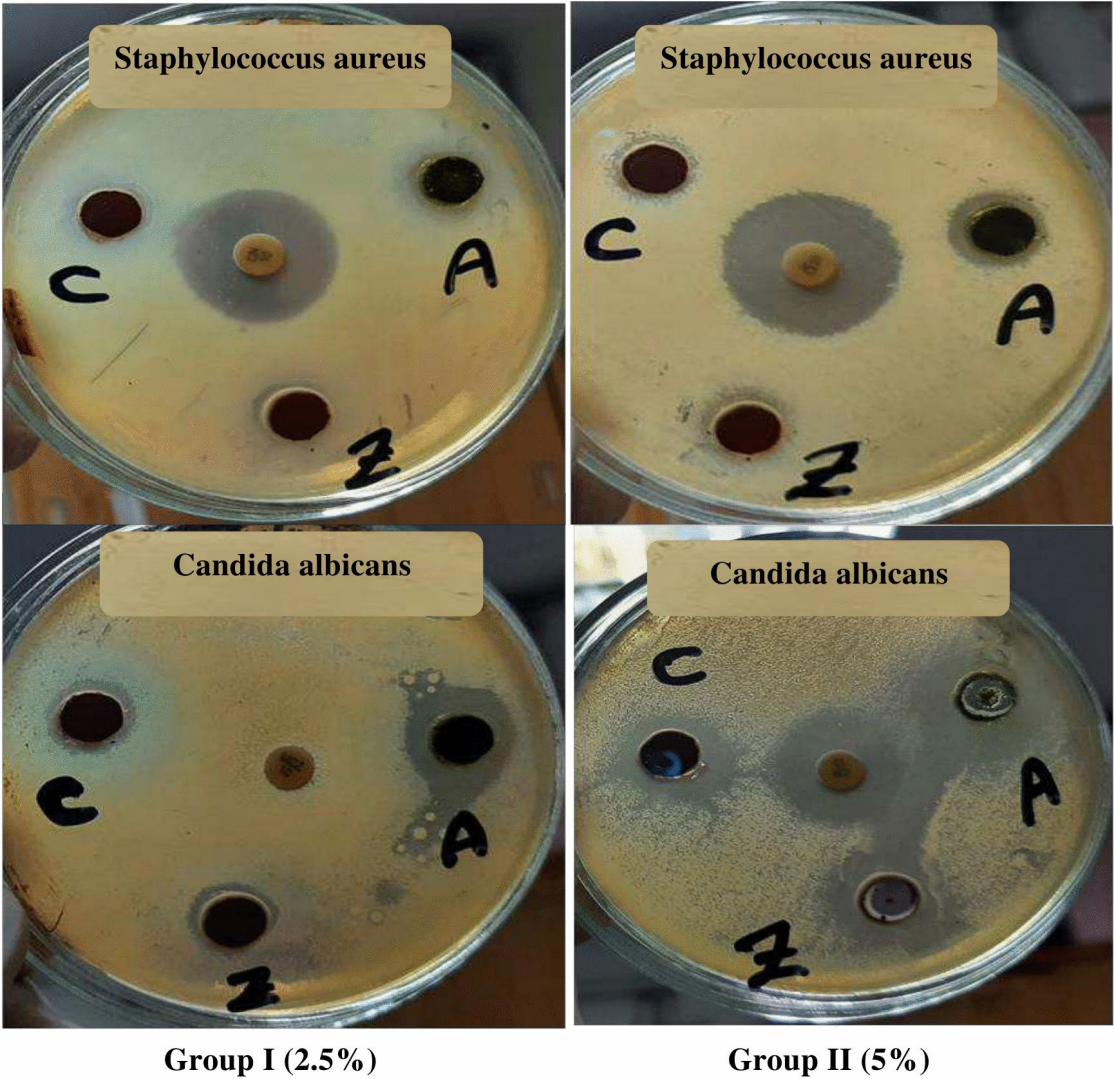
Inhibition zone of coatings containing algal biomass (A), zinc ferrite/AB (Z) and cerium ferrite/AB (C) in both groups.

Table 3 and Fig. 14 present the antimicrobial activity of the coatings containing both algal biomass and nanopigments. The coatings containing algal biomass and nanopigments have inhibitory effects against both bacterial strains such as *Staphylococcus aureus* and fungal strains such as *Candida albicans*. Also, a larger diameter of the inhibition zone is observed for discs in group II that contain 5%. There is no significant difference between the inhibition zone diameters of zinc ferrite/AB and cerium ferrite/AB.

**Table 3:** Antimicrobial activity of the prepared coatings.

Strains	Nano-pigments		
	Group I (2.5%)		
	Algal biomass	Zinc ferrite/AB	Cerium ferrite/AB
*Candida albican*	21 ± 0.041	16 ± 0.077	17 ± 0.02
*Staphylococcus aureus*	16 ± 0.02	15 ± 0.036	15 ± 0.05

Strains	Nano-pigments		
	Group II (5%)		
	Algal biomass	Zinc ferrite/AB	Cerium ferrite/AB
*Candida albican*	23 ± 0.08	21 ± 0.01	21 ± 0.051
*Staphylococcus aureus*	18 ± 0.02	16 ± 0.064	15 ± 0.02

**Fig. 15:**
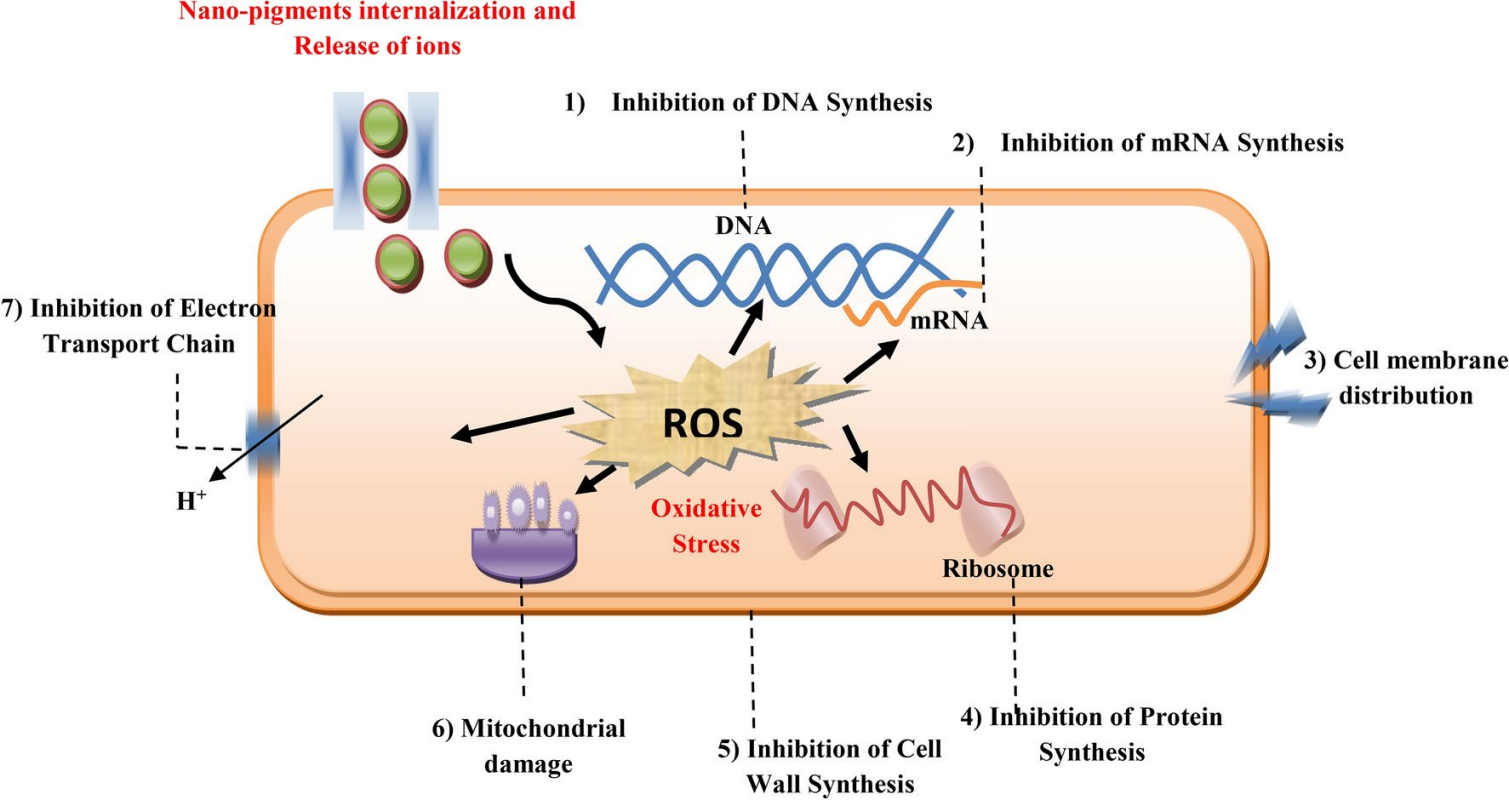
Mechanism of nanopigments microbial toxicity.

It is well known that algae bio-products are among the most valuable antimicrobial compounds due to the existence of proteins, peptides, polysaccharides, polyphenols, fatty acids, and pigments. Furthermore, some studies propose that the production of reactive oxygen species (ROS), which ultimately results in bacterial death as depicted in Fig. 15^60^, is the cause of the inhibitory activity produced by nanoparticles (NPs). Findings by Arakha et al.^61^ demonstrated that the release of ROS upon contact with iron oxide (NPs) is the antimicrobial mechanism responsible for bacterial death. When NPs with both positive and negative surface charges were examined, those with a positive charge exhibited more powerful antimicrobial activity. Oxidative stress is a result of the processes produced by photocatalytic activities in bacteria. These ultimately experience a rupture of the cell membrane that allows cytoplasmic materials to seep out^62^. The attraction of nanoparticles to the surface of bacteria as a result of load variations is one of the other potential processes. The negative surface charge of bacteria attracts metal cations, which build up and allow entry into the cell through gaps in the wall or membrane and intracellular content loss^63^.

Moreover, ZnO is a transition metal oxide and semiconductor. ZnO has a broad band gap of 3.3 eV. Electron–hole pairs arise when the radiation’s energy exceeds the band gap of ZnO. The conduction band is opened up to electrons. Extremely oxidizing sites that can oxidize water molecules or hydroxide anions and produce strong oxidizing species are developed as a result of the extremely oxidizing nature of the hole that is produced in the valence band. As a result of this reaction, reactive oxygen species (ROS) are produced, with hydroxyl radical ($O{H}^{\cdot }$), hydroperoxyde radical ($H{{O}^{\cdot }}_{2}^{-}$) and superoxide radical anion $\left({{O}^{\cdot }}_{2}^{-}\right)$ serving as the routes for bactericidal activity. The good antimicrobial activity of coatings based on nano-ZnO may be due to its wide band gap. The following chemical equations describe how ROS is generated for ZnO.

$$\begin{aligned} & {\text{O}}_{{2}} + {\text{ e}}^{-} \to {\text{O}}_{{2}}^{-} \\ & {\text{H}}_{{2}} {\text{O }} + {\text{ h}}^{ + } \to {\text{OH}}^{-} + {\text{ H}}^{ + } \\ & {\text{OH}}^{ \cdot } + {\text{ OH}}^{ \cdot } \to {\text{H}}_{{2}} {\text{O}}_{{2}} \\ & {\text{O}}_{{2}}^{ - } + {\text{ H}}_{{2}} {\text{O}}_{{2}} \to {\text{OH}}^{ - } + {\text{ OH}}^{ - } + {\text{ O}}_{{2}} \\ & {\text{OH}}^{ \cdot } \, + {\text{ O}}_{{2}} + {\text{ organic}} \to {\text{CO}}_{{2}} + {\text{ H}}_{{2}} {\text{O}} \\ & \quad \quad \quad \downarrow \\ & {\text{Cell membrane of microorganism}} \\ \end{aligned}$$

On the other hand, *Candida albicans* is a strong fungus that can easily resist many antifungals due to its adaptability and ability to establish resistance through frequent contact with antifungals. Some of the factors that can lead to antifungal tolerance and resistance include the ability to evade host immune defenses, the formation of biofilms, which reduces the accessibility of the antifungal, the selection of spontaneous mutations that boost expression or reduce the susceptibility of the *Candida* species, altered chromosome abnormalities, overexpression of multidrug efflux pumps, and altered chromosome abnormalities^61^. However, the results reveal that *Candida albicans* is very sensitive to the prepared nano-pigments, so these pigments can be identified as very strong antifungal agents^63–65^.

### Investigation of thermal stability

#### Thermal gravimetric analysis (TGA)

**Fig. 16:**
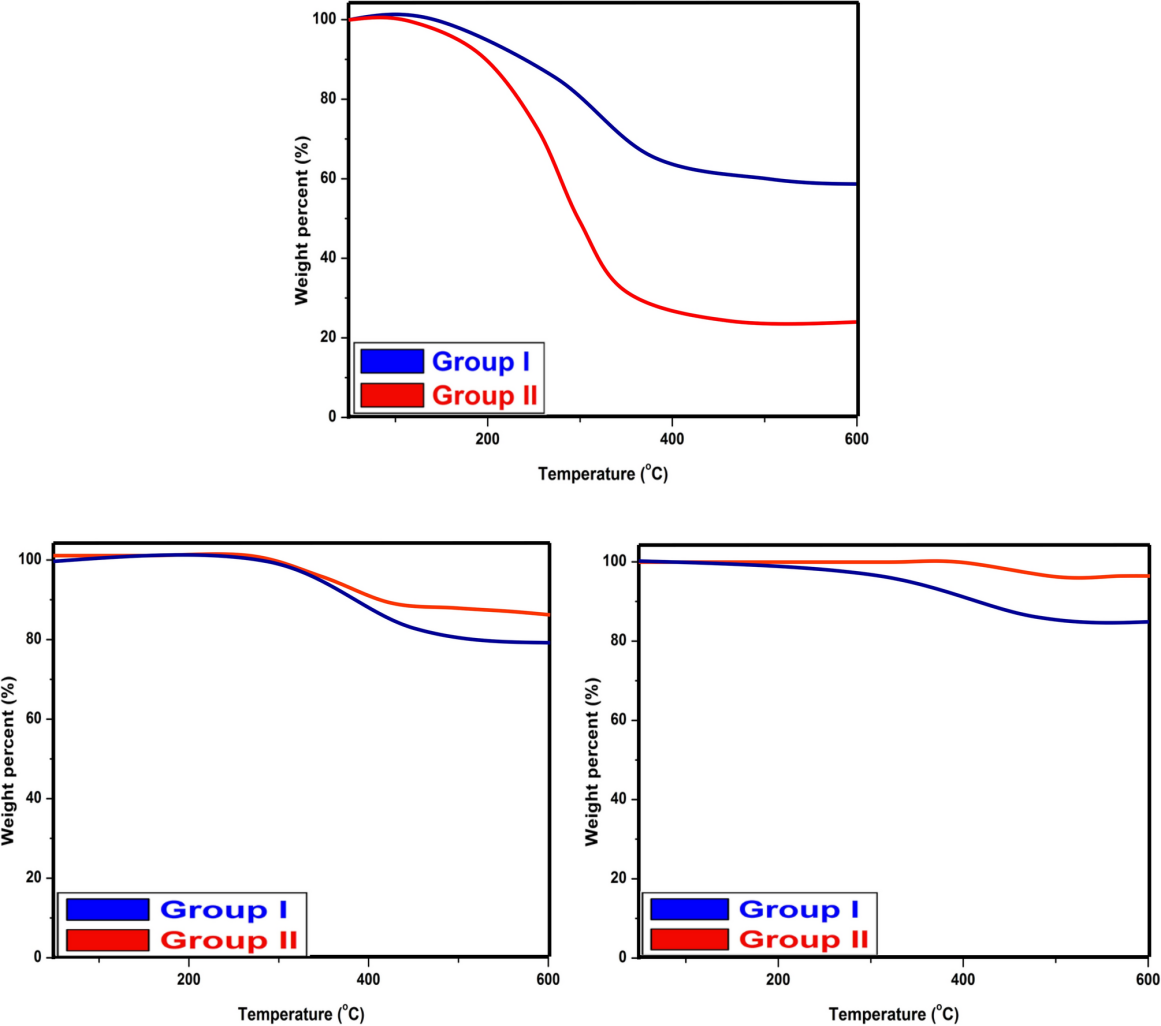
TGA of coatings containing algal biomass, zinc ferrite/AB and cerium ferrite/AB in both groups.

Figure 16 clarifies the TGA results of films containing coatings containing both algal biomass and nanopigments. The results of the paint film containing algal biomass showed very high weight loss, reaching 47.5 in group I and 76% in group II. Thus, the thermal stability of the coatings containing algal biomass is decreased by increasing its concentration. This may be attributed to the evaporation of water in the internal cells, the production of carbon oxides, and the pyrolysis process that occurred during the decomposition of algal biomass. Besides, most of the volatile organic compounds are released, as mentioned above in the TGA explanation of algal biomass.

In the case of coatings containing zinc ferrite/AB and cerium ferrite/AB, weight loss doesn’t exceed 20%, and films containing 5% are the best. Additionally, the findings demonstrate that weight loss is decreased by increasing the concentration of the prepared nanopigments due to close connections between molecules^66^. The low weight loss noticed in the coatings containing zinc ferrite/AB and cerium ferrite/AB at almost 350 °C is attributed to the breakdown of the alkyd network due to the cleavage of the C–O bond^67^.

#### Scanning electron microscopy (SEM) of the coated films exposed at 525 °C

**Fig. 17:**
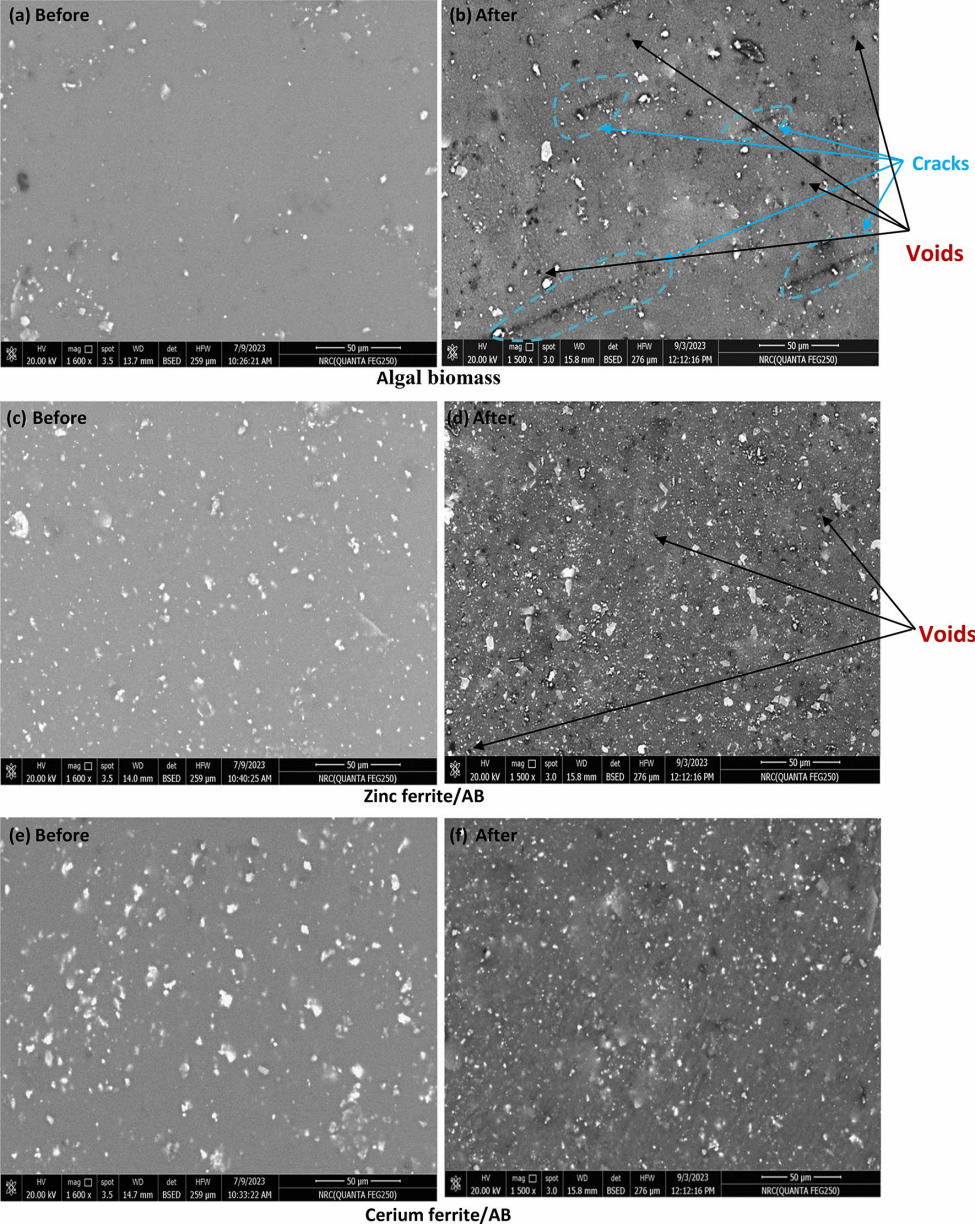
SEM of coatings before and after exposure to high temperatures in group I.

**Fig. 18:**
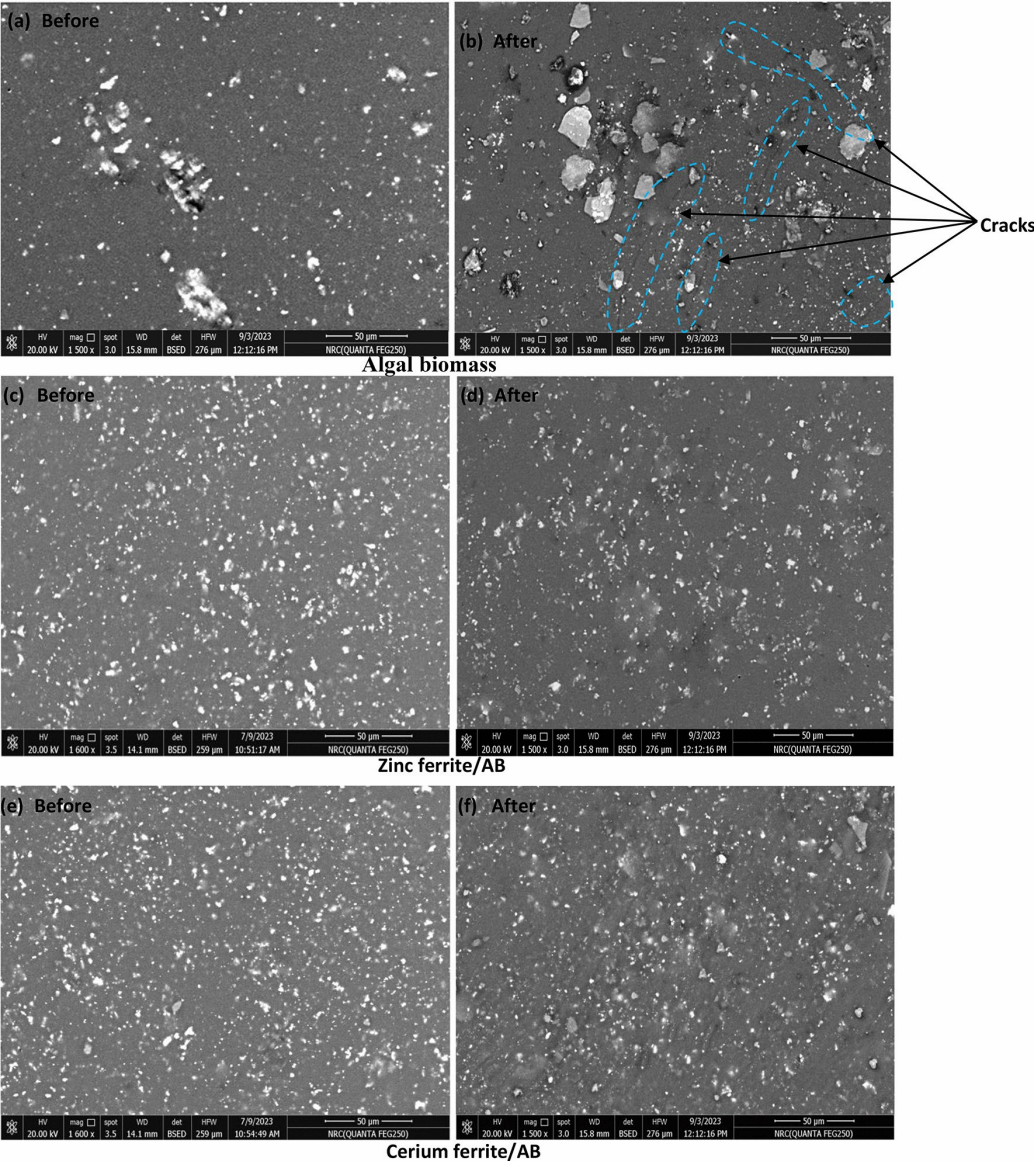
SEM of coatings before and after exposure to high temperatures in group II.

Figures 17, 18 exhibit the SEM photos of films including both algal biomass and nanopigments. In the image of films with algal biomass in groups I and II, cracks and agglomerations are observed after exposure to high temperatures, which means that their heat resistance is very low and they are affected by the elevated temperature. The images of films including zinc ferrite/AB and cerium ferrite/AB in group I show the appearance of voids, and this could be related to the low concentration of nanopigments in the polymeric matrix. In group II, the films containing both nanopigmenst present a uniform surface with good distribution and without cracking or voids. The absence of cracking or voids and the good adhesion mean that the coating is thermally stable; this proves that the naturally sourced nanopigments could promote the heat resistance of the alkyd resin^68,69^.

## Conclusions

Algal biomass was utilized to create new, cost-effective, and sustainable nanopigments characterized by vibrant colors, thermal stability, and antimicrobial properties, suitable for the coatings industry. Algal cells, grown in wastewater, were harvested, dried, and converted into biomass, then encapsulated with a thin nano-layer of zinc ferrite or cerium ferrite. The resulting nanopigments were incorporated into alkyd resin at two concentrations, 2.5% and 5%. Coatings incorporating algal biomass and naturally sourced nanopigments exhibit significant antimicrobial properties against *Staphylococcus aureus* and *Candida albicans*, with the 5% formulation demonstrating the strongest inhibition. The thermal stability results of the film containing algal biomass show very high weight loss, reaching 47.5% in group I and 76% in group II. While in the case of coatings containing zinc ferrite/AB and cerium ferrite/AB, weight loss doesn’t exceed 20%, and films containing 5% are the best. The color results declare that the films containing green algal biomass have (−a) values due to their green color. In the case of films containing both zinc ferrite/AB and cerium ferrite/AB, it was found that the (+ a) values are observed due to the red color. Finally, it could be concluded that utilizing algal biomass sourced from wastewater presents an economically viable option for the coatings industry, while the resulting nanopigments are more sustainable than traditional alternatives. These innovative paints are suitable for a range of applications, including biomedical device coatings requiring antimicrobial effects, high-temperature furnaces, and decorative uses due to their bright color and gloss.

## Data Availability

Data will be made available on request from the corresponding author email: sayedamohammed2015@mail.com.
